# The influence of long-term use of proton pump inhibitors on the gut microbiota: an age-sex-matched case-control study

**DOI:** 10.3164/jcbn.17-78

**Published:** 2017-12-12

**Authors:** Tomohisa Takagi, Yuji Naito, Ryo Inoue, Saori Kashiwagi, Kazuhiko Uchiyama, Katsura Mizushima, Saeko Tsuchiya, Tetsuya Okayama, Osamu Dohi, Naohisa Yoshida, Kazuhiro Kamada, Takeshi Ishikawa, Osamu Handa, Hideyuki Konishi, Kayo Okuda, Yoshimasa Tsujimoto, Hiromu Ohnogi, Yoshito Itoh

**Affiliations:** 1 Molecular Gastroenterology and Hepatology, Graduate School of Medical Science, Kyoto Prefectural University of Medicine, 465 Kajii-cho, Kamigyo-ku, Kyoto 602-8566, Japan; 2 Department for Medical Innovation and Translational Medical Science, Graduate School of Medical Science, Kyoto Prefectural University of Medicine, 465 Kajii-cho, Kamigyo-ku, Kyoto 602-8566, Japan; 3 Laboratory of Animal Science, Kyoto Prefectural University, Sakyo-ku, Kyoto 606-8522, Japan; 4 Takara Bio Inc., Nojihigashi 7-4-38, Kusatsu, Shiga 525-0058, Japan

**Keywords:** proton pump inhibitors (PPIs), gut microbiota, 16S rRNA

## Abstract

Proton pump inhibitors (PPIs) are widely used to treat gastro-esophageal reflux and prevent gastric ulcers, and have been considered as low risk. However, recent studies have identified possible associations between PPI use and gut microbiota, suggesting that PPIs use increases the risk of enteric infections, including *Clostridium difficile* infection. To investigate gut microbiota in Japanese PPIs users, we conducted 16S metagenomics analysis of fecal samples collected from PPI users and healthy adults. In total, 36 PPI users and 36 PPI non-users (as control subjects) matched by age and sex were recruited and fecal samples were obtained to analyze the gut microbiome using 16S rRNA gene sequencing. There were significant differences in the microbial structure between PPI non-users and PPI users. In contrast, the analysis of α-diversity revealed no significant differences between PPI non-users and PPI users. When comparing in genus level between these two groups, the genera *Streptococcus* was significantly abundant and the genera *Faecalibacterium* was significantly decreased in PPI users. Our findings indicate a probable association between PPI use and the alternation of microbiota. These alterations might provide a mechanism by which PPIs predispose enteric infection such as *Clostridium difficile* infection.

## Introduction

Proton pump inhibitors (PPIs), which work by blocking the proton pump that transports acid into the stomach, are used worldwide to treat heartburn and other symptoms caused by gastroesophageal reflux disease (GERD) and functional dyspepsia (FD). In addition, PPIs are used to treat or prevent gastroduodenal peptic ulcers and injuries associated with the use of nonsteroidal anti-inflammatory drugs (NSAIDs) and aspirin. However, recent literature suggests that long-term PPI use may be associated with a variety of adverse events, including bone metabolism, fracture, hypomagnesaemia, vitamin B12 deficiency, iron deficiency anemia, and pneumonia.^(1,2)^

In addition, recent reports demonstrated that the composition of gut microbiota in PPI users is altered.^(3,4)^ As recent studies have revealed the composition of the human gut microbiota has been linked to host health and to the development of diseases,^(5)^ the use of PPIs may be associated with an increased risk of enteric infections including *Clostridium difficile* infection (CDI).^(6,7)^ Therefore, uncovering the alternation of the gut microbiota due to PPI administration may be an important issue. We aimed to evaluate the microbiome associated with long-term PPI use in Japanese individuals.

## Materials and Methods

### Ethics statements

This study conformed to the code of ethics stated in the Declaration of Helsinki. The Ethics Committee of Kyoto Prefectural University of Medicine approved the research protocol (permission No. ERB-C-534), and all participants provided written informed consent prior to enrollment. The study was registered at the University Hospital Medical Information Network Center (UMIN 000019486).

### Patients

Seventy-two patients were prospectively selected from our outpatient clinic from November 2016 to April 2017. There were 36 PPI users and 36 PPI non-users as control subjects matched by age and sex, and details of the study subjects are summarized in Table 1. Persons with PPI administration over a period of at least 1 year prior to study enrollment were eligible to be enrolled as PPI users. Persons who had not been dispensed PPIs in the 5 years prior to study enrollment were considered PPI non-users.

### Sample collection and DNA extraction

The fecal samples, the size of a grain of rice, were collected using a feces collection kit (Techno Suruga Lab, Shizuoka, Japan). After vigorous mixing, the samples were stored at a temperature not higher than a room temperature for 7 days until DNA extraction.

Genomic DNA was isolated using the NucleoSpin Microbial DNA Kit (MACHEREY-NAGEL, Düren, Germany). Approximately 500 µl of stored fecal samples were placed into a microcentrifuge tube containing 100 µl Elution Buffer BE. The mixture was then placed into the NucleoSpin Beads Tube with Proteinase K, which was subjected to mechanical beads-beating for 12 min at 30 Hz in the TissueLyzer LT. The subsequent extraction procedure was performed per the manufacturer’s instructions. Extracted DNA samples were purified using the Agencourt AMPure XP (Beckman Coulter, Brea, CA).

### Sequencing of 16S rRNA gene

Two step PCRs were performed for the purified DNA samples to obtain sequence libraries. The first PCR was performed to amplify using a 16S (V3–V4) Metagenomic Library Construction Kit for NGS (Takara Bio Inc., Kusatsu, Japan) with primer pairs of 341F (5'-TCGTCGGCAGCG TCAGATGTGTATAAGAGACAGCCTACGGGNGGCWGCAG-3') and 806R (5'-GTCTCGTGGGCTCGGAGATGTGTATAAGAG ACAGGGACTACHVGGGTWTCTAAT-3') corresponding to the V3–V4 region of the 16S rRNA gene. The second PCR was done to add the index sequences for Illumina sequencer with barcode sequence using the Nextera XT Index kit (Illumina, San Diego, CA). The prepared libraries were subjected to the sequencing of paired-end 300 bases using the MiSeq Reagent Kit v3 on the MiSeq (Illumina) at the Biomedical Center, Takara Bio.

### Microbiome analysis

Processing of sequence data, including chimera check, operational taxonomic unit (OTU) definition, and taxonomy assignment was performed using QIIME ver. 1.9,^(8)^ USEARCH ver. 8.0,^(9)^ and UCHIME ver. 4.2.40^(10)^ according to Inoue *et al.*^(11)^ respectively. Singletons were removed in this study. Taxonomy assignment of the resulting OTU was completed using RDP classifier ver. 2.10.2 with the Greengenes database (published May 2013). Statistical differences (*p*<0.05) in the relative abundance of bacterial phyla and genera between groups were evaluated using Student’s paired *t* tests.

The observed species, Chao1 and Shannon phylogenetic diversity indices, were calculated by the R “phyloseq” package and were statistically analyzed using a Wilcoxon’s rank sum test. β-diversity was estimated using the UniFrac metric to calculate the distances between the samples and visualized by principal coordinate analysis (PCoA), and was statistically examined using permutational multivariate analysis of variance (PERMANOVA). The final figures were generated using the software QIIME (ver. 1.9.0).

Potential changes in the microbiome at the functional level were evaluated using PICRUSt software,^(12)^ and the Kyoto Encyclopedia of Genes and Genomes (KEGG) database, release 70.0.^(13)^ The human-specific pathways were removed from the results to focus on true bacterial pathways. The PICRUSt software uses 16S-rRNA sequence profiles to estimate metagenome content based on reference bacterial genomes and the KEGG pathway database. The result was further statistically analyzed by Welch’s *t* test using the STAMP software.^(14)^ *P *values (<0.05) were used to determine the statistical significance between the groups.

## Results

**Fig. 1:**
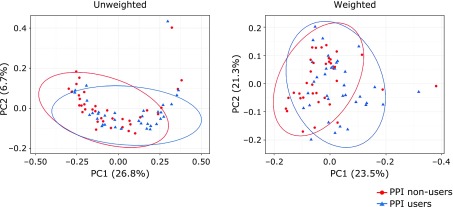
Principal coordinate analysis (PCoA) plots of PPI non-users vs PPI users. Distances were calculated with unweighted and weighted UniFrac. Distances between PPI non-users and PPI users were significantly different (*p*<0.05 with PERMANOVA tests).

**Fig. 2:**
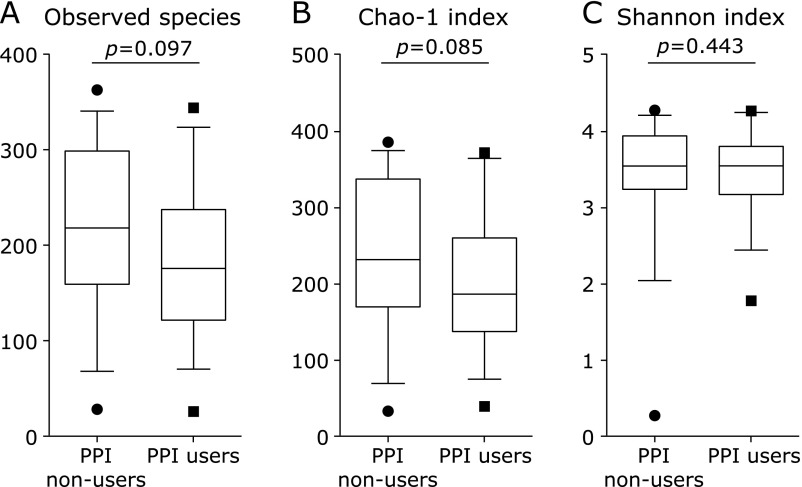
The α-diversity indices between PPI non-users and PPI users. We compared α-diversity indices [the observed species and the Chao 1 index (OTU richness estimation), and the Shannon index (OTU evenness estimation)] using Student’s unpaired *t* tests.

Eight-hundred eight OTUs were detected in this study. Initially, the overall structure of the gut microbiome between PPI non-users and PPI users using β-diversity indices was calculated for unweighted and weighted UniFrac distance (Fig. 1). The principal coordinate analysis (PCoA) revealed that there were microbial structural differences between PPI non-users and PPI users in unweighted (PERMANOVA, *p*<0.02) and weighted (PERMANOVA, *p*<0.01) UniFrac distances. Subsequently, we compared α-diversity between PPI non-users and PPI users using different indices [the observed species and the Chao 1 index (OTU richness estimation), and the Shannon index (OTU evenness estimation) (Fig. 2). Although the observed species and the Chao 1 index showed a decreasing trend in PPI users group, there was no statistically significant difference in α-diversity between these two groups.

**Fig. 3:**
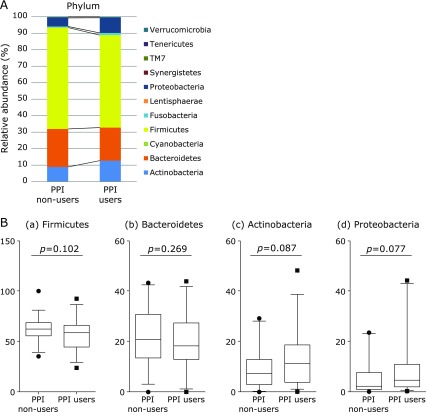
Comparative analyses of the taxonomic composition of the microbial community at the phylum level. (A) Each component of the cumulative bar chart indicates a phylum. (B) The representative phyla (Firmicutes, Bacteroidetes, Proteobacteria and Actinobacteria) were evaluated between PPI non-users and PPI users using Student’s unpaired *t* tests.

The differences in the gut microbial structure were taxonomically evaluated at the phylum level (Fig. 3A). In the abundance of the phylum Firmicutes and Bacteroidetes, there was no significant difference between PPI non-users and PPI users [Fig. 3B (a and b)]. In contrast, the abundance of the phylum Proteobacteria was considerably higher in the PPI users group, though the difference was not statistically significant [Fig. 3B (c)]. The abundance of the phylum Actinobacteria also showed an increasing trend in the PPI users group, though the difference was not statistically significant [Fig. 3B (d)].

**Fig. 4:**
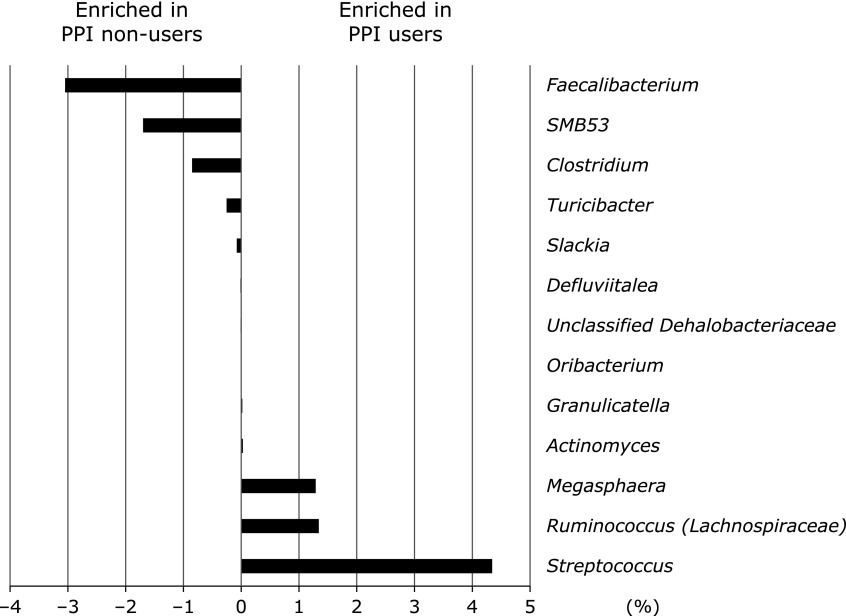
Comparative analyses of the taxonomic composition of the microbial community at the genus level. The significant different genera between PPI non-users and PPI users were presented.

The taxonomic changes in the microbial community were evaluated at the genus levels. As shown in Fig. 4 and Supplemental Table 1*, the comparison of the microbial changes between PPI non-users and PPI users showed a significant decrease in the abundance of 8 genera and a significant increase in 5 genera in PPI users. These were characterized by a decrease in the abundance of the genera *Faecalibacterium* (*p*<0.01), *SMB53* (*p*<0.01), *Clostridium* (*p*<0.01), *Turicibacter* (*p*<0.05), *Slackia* (*p*<0.01), *Defluviitalea* (*p*<0.05), Unclassified *Dehalobacteriaceae* (*p*<0.05), and *Oribacterium* (*p*<0.05), and by an increase in the abundance of the genera *Streptococcus* (*p*<0.01), *Ruminococcus* (*Lachnospiraceae*) (*p*<0.05), *Megasphaera* (*p*<0.05), *Actinomyces* (*p*<0.05), and *Granulicatella* (*p*<0.05).

**Fig. 5:**
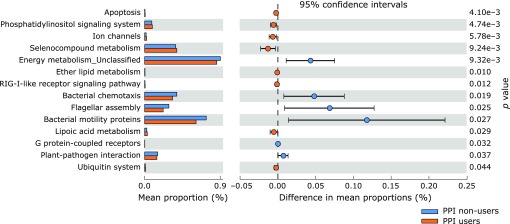
The relative abundance of functional pathways in gut microbiota between PPI non-users and PPI users. The KEGG database functional categories are shown with the displayed histograms (left panel: means) and q-value determinations (right panel: 95% confidence intervals).

Potential differences in the function of the microbiome were evaluated using PICRUSt software (Fig. 5).^(12)^ When comparing PPI users with PPI non-users, the proportion of genes responsible for apoptosis, phosphatidylinositol signaling, ion channels, selenocompound metabolism, ether lipid metabolism, RIG-I-like receptor signaling, lipoic acid metabolism, and ubiquitin system was significantly increased in PPI users.

## Discussion

In the present study, we described the differences of gut microbiota between PPI non-users (as control subjects) and PPI users in Japanese individuals. First, using the unweighted and weighted UniFrac distance, we compared the overall microbial structure between PPI non-users and PPI users. Importantly, as shown in Fig. 1, the unweighted and weighted PCoA indicated significant structural differences between these two groups. Thus, an obvious shift of the PPI users away from the PPI non-user cluster in the composition of the gut microbiota was observed. Similar to our results, Tsuda *et al.*^(15)^ and Imhann *et al.*^(4)^ previously showed the microbiota of PPI users were different from PPI non-users. Conversely, Freedberg *et al.*^(3)^ reported that the gut microbiota between PPI non-users and PPI users did not alter, though their investigation based on short-term (4 weeks) PPI administration.

Regarding α-diversity indices (microbiota diversity), Clooney *et al.*^(16)^ found that α-diversity is no different between long-term PPI users (more than 5 years) and PPI non-users. On the other hand, two studies from large cohorts have demonstrated a significant decrease of α-diversity in PPI users.^(4,17)^ In our results, as shown in Fig. 2, α-diversity indices (microbiota diversity) between PPI non-users and PPI users revealed no significant differences, though there appears to be a lower distribution of diversities within PPI users.

In addition, we could not find taxonomically evaluated changes at the phylum level. One of the most striking results was an increase in the abundance of the genera *Streptococcus* and a decrease in the abundance of the genera *Faecalibacterium*. Consistent with our results, most investigations revealed PPI administration increased the genera *Streptococcus*.^(3,15–17)^ Interestingly, it has also been shown that potassium-competitive acid blockers, which have more potent gastric acid suppression than PPIs, caused an increase in the genus *Streptococcus* compared with PPI.^(18)^ Additionally, Tsuda *et al.*^(15)^ demonstrated that the abundance of *Faecalibacterium* was also lower in PPI users compared with that in PPI non-users. Garcia-Mazcorro *et al.*^(19)^ demonstrated that omeprazole decreased *Faecalibacterium* in healthy male dogs. Furthermore, it is well-known that *Faecalibacterium* is one of the most abundant anaerobic bacteria in the human gut, and plays an important role in providing, not only energy to the colonocytes and maintaining the intestinal health, but also an anti-inflammatory effect.^(20)^ In fact, the decrease of *Faecalibacterium* was observed in patients with inflammatory bowel diseases (IBD), including ulcerative colitis and Crohn’s disease.^(21–23)^. Thus, the decrease of *Faecalibacterium *by PPI use might be associated with intestinal inflammation.

Using computational prediction from 16S rRNA metagenomic data, this study represents the gut microbiota-associated functional profiles in PPI users in Japanese individuals (Fig. 5). Although these findings might highlight the gut microbial functional pathways possibly implicated in PPI users, more studies are needed to understand the significance and the role of the gut microbial functional pathways.

In summary, the present study indicated that treatment with PPI alerted the gut microbiota, characterized by an increase in the abundance of the genera *Streptococcus* and a decrease in the abundance of the genera *Faecalibacterium*. Further studies are warranted to elucidate the mechanisms involved in this phenomenon and ensure complete awareness of the influence of long-term PPI use on the gut microbiome.

## Figures and Tables

**Table 1:** Baseline characteristics of enrolled patients

	PPI non-users (*n* = 36)	PPI users (*n* = 36)
Male/Female	22/14	22/14
Age (median)	74 (48–85)	74 (48–85)
PPI		
Esomeprazol	-	8
Lansoprazol	-	16
Rabeprazol	-	11
Omeprazol	-	1
PPI indications		
GERD	-	2
Functional dyspepsia	-	2
Ulcer treatment	-	6
Ulcer prevention	-	22
Others	-	4
