# Supplementary material for: The influence of long-term use of proton pump inhibitors on the gut microbiota: an age-sex-matched case-control study
Source: J Clin Biochem Nutr. 2017 Dec 12;62(1):100–5. doi: 10.3164/jcbn.17-78 (PMC5773837; doi:10.3164/jcbn.17-78)
Supplement: Supplemental Table 1 [file jcbn17-78st01.pdf]

**Supplemental Table 1.** Baseline characteristics of enrolled patients

| Phylum | Family | Genus | PPI non-users (%) | PPI users (%) |
|---|---|---|---|---|
| Euryarchaeota | Methanobacteriaceae | Methanobrevibacter | 0.023 ± 0.0831 | 0.0191 ± 0.0976 |
| Other | Other | Other | 0.000 ± 0.0005 | 0.0000 ± 0.0000 |
| Actinobacteria | Actinomycetaceae | Unclassified | 0.000 ± 0.0012 | 0.0008 ± 0.0019 |
| Actinobacteria | Actinomycetaceae | Actinomyces* | 0.023 ± 0.0225 | 0.0505 ± 0.0587 |
| Actinobacteria | Corynebacteriaceae | Corynebacterium | 0.002 ± 0.0031 | 0.0018 ± 0.0029 |
| Actinobacteria | Microbacteriaceae | Microbacterium | 0.000 ± 0.0000 | 0.0005 ± 0.0030 |
| Actinobacteria | Micrococcaceae | Micrococcus | 0.000 ± 0.0000 | 0.0003 ± 0.0013 |
| Actinobacteria | Micrococcaceae | Rothia | 0.010 ± 0.0176 | 0.0374 ± 0.0917 |
| Actinobacteria | Bifidobacteriaceae | Unclassified | 0.002 ± 0.0132 | 0.0000 ± 0.0000 |
| Actinobacteria | Bifidobacteriaceae | Alloscardovia | 0.001 ± 0.0081 | 0.0052 ± 0.0142 |
| Actinobacteria | Bifidobacteriaceae | Bifidobacterium | 6.089 ± 6.6199 | 9.3297 ± 11.0353 |
| Actinobacteria | Coriobacteriaceae | Other | 0.007 ± 0.0262 | 0.0000 ± 0.0000 |
| Actinobacteria | Coriobacteriaceae | Unclassified | 0.153 ± 0.1748 | 0.1391 ± 0.2381 |
| Actinobacteria | Coriobacteriaceae | Adlercreutzia | 0.060 ± 0.0746 | 0.0347 ± 0.0765 |
| Actinobacteria | Coriobacteriaceae | Atopobium | 0.007 ± 0.0103 | 0.0054 ± 0.0083 |
| Actinobacteria | Coriobacteriaceae | Collinsella | 2.385 ± 2.2653 | 2.9625 ± 2.7286 |
| Actinobacteria | Coriobacteriaceae | Coriobacterium | 0.008 ± 0.0223 | 0.0042 ± 0.0104 |
| Actinobacteria | Coriobacteriaceae | Eggerthella | 0.064 ± 0.0834 | 0.1526 ± 0.3354 |
| Actinobacteria | Coriobacteriaceae | Enterococcus | 0.004 ± 0.0077 | 0.0020 ± 0.0046 |
| Actinobacteria | Coriobacteriaceae | Slackia* | 0.098 ± 0.1727 | 0.0221 ± 0.0586 |
| Bacteroidetes | Other | Other | 0.010 ± 0.0258 | 0.0029 ± 0.0089 |
| Bacteroidetes | Unclassified | Unclassified | 0.036 ± 0.2133 | 0.0000 ± 0.0000 |
| Bacteroidetes | Bacteroidaceae | Bacteroides | 13.047 ± 9.5484 | 13.2324 ± 8.8957 |
| Bacteroidetes | Porphyromonadaceae | Parabacteroides | 1.525 ± 1.4380 | 1.7686 ± 1.6104 |
| Bacteroidetes | Porphyromonadaceae | Porphyromonas | 0.005 ± 0.0281 | 0.0000 ± 0.0000 |
| Bacteroidetes | Prevotellaceae | Unclassified | 0.011 ± 0.0542 | 0.0000 ± 0.0000 |
| Bacteroidetes | Prevotellaceae | Prevotella | 6.058 ± 10.9595 | 3.2364 ± 7.5336 |
| Bacteroidetes | Rikenellaceae | Unclassified | 1.093 ± 1.4838 | 1.0382 ± 1.2732 |
| Bacteroidetes | Rikenellaceae | Alistipes | 0.051 ± 0.0842 | 0.1045 ± 0.2638 |
| Bacteroidetes | S24-7 | Unclassified | 0.101 ± 0.2484 | 0.0226 ± 0.0670 |
| Bacteroidetes | [Barnesiellaceae] | Unclassified | 0.301 ± 0.5325 | 0.2080 ± 0.3745 |
| Bacteroidetes | [Odoribacteraceae] | Butyricimonas | 0.091 ± 0.1302 | 0.1430 ± 0.2102 |
| Bacteroidetes | [Odoribacteraceae] | Odoribacter | 0.139 ± 0.2023 | 0.0921 ± 0.1091 |
| Bacteroidetes | [Paraprevotellaceae] | Unclassified | 0.023 ± 0.1234 | 0.0001 ± 0.0007 |
| Bacteroidetes | [Paraprevotellaceae] | CF231 | 0.016 ± 0.0704 | 0.0000 ± 0.0000 |
| Bacteroidetes | [Paraprevotellaceae] | Paraprevotella | 0.367 ± 0.7437 | 0.1740 ± 0.3146 |
| Bacteroidetes | [Paraprevotellaceae] | [Prevotella] | 0.171 ± 0.9421 | 0.0023 ± 0.0140 |
| Bacteroidetes | Sphingobacteriaceae | Solitalea | 0.002 ± 0.0094 | 0.0000 ± 0.0000 |
| Cyanobacteria | Unclassified | Unclassified | 0.014 ± 0.0774 | 0.0178 ± 0.1066 |
| Firmicutes | Bacillaceae | Bacillus | 0.204 ± 0.4177 | 0.0663 ± 0.2506 |
| Firmicutes | Staphylococcaceae | Staphylococcus | 0.000 ± 0.0006 | 0.0036 ± 0.0168 |
| Firmicutes | Gemellaceae | Unclassified | 0.003 ± 0.0045 | 0.0088 ± 0.0175 |
| Firmicutes | Unclassified | Unclassified | 0.001 ± 0.0062 | 0.0208 ± 0.0817 |
| Firmicutes | Aerococcaceae | Abiotrophia | 0.001 ± 0.0017 | 0.0008 ± 0.0017 |
| Firmicutes | Carnobacteriaceae | Carnobacterium | 0.000 ± 0.0000 | 0.0004 ± 0.0026 |
| Firmicutes | Carnobacteriaceae | Granulicatella** | 0.008 ± 0.0085 | 0.0269 ± 0.0342 |
| Firmicutes | Enterococcaceae | Enterococcus | 2.924 ± 16.4669 | 0.2004 ± 0.5374 |
| Firmicutes | Lactobacillaceae | Unclassified | 0.008 ± 0.0363 | 0.0009 ± 0.0024 |
| Firmicutes | Lactobacillaceae | Lactobacillus | 0.994 ± 4.5451 | 2.2922 ± 5.0451 |
| Firmicutes | Leuconostocaceae | Leuconostoc | 0.000 ± 0.0003 | 0.0006 ± 0.0032 |
| Firmicutes | Streptococcaceae | Lactococcus | 0.004 ± 0.0201 | 0.0005 ± 0.0016 |
| Firmicutes | Streptococcaceae | Streptococcus** | 1.612 ± 2.3494 | 5.9562 ± 5.4111 |
| Firmicutes | Turicibacteraceae | Turicibacter* | 0.293 ± 0.6337 | 0.0393 ± 0.1564 |
| Firmicutes | Other | Other | 0.160 ± 0.4284 | 0.0718 ± 0.1491 |
| Firmicutes | Unclassified | Unclassified | 1.425 ± 2.8448 | 0.5700 ± 0.8660 |
| Firmicutes | Christensenellaceae | Other | 0.010 ± 0.0203 | 0.0039 ± 0.0088 |
| Firmicutes | Christensenellaceae | Unclassified | 1.017 ± 3.4062 | 0.2399 ± 0.5615 |

| Phylum | Family | Genus | PPI non-users (%) | PPI users (%) |
|---|---|---|---|---|
| Firmicutes | Christensenellaceae | Christensenella | 0.001 ± 0.0027 | 0.0022 ± 0.0054 |
| Firmicutes | Clostridiaceae | Unclassified | 0.017 ± 0.0466 | 0.0115 ± 0.0600 |
| Firmicutes | Clostridiaceae | Clostridium** | 1.220 ± 1.4848 | 0.3701 ± 0.5130 |
| Firmicutes | Clostridiaceae | SMB53** | 2.297 ± 2.4217 | 0.5988 ± 0.8915 |
| Firmicutes | Clostridiaceae | Sarcina | 0.012 ± 0.0315 | 0.0547 ± 0.3002 |
| Firmicutes | Dehalobacteriaceae | Unclassified* | 0.010 ± 0.0253 | 0.0003 ± 0.0014 |
| Firmicutes | Dehalobacteriaceae | Dehalobacterium | 0.027 ± 0.1163 | 0.0045 ± 0.0123 |
| Firmicutes | Eubacteriaceae | Anaerofustis | 0.002 ± 0.0048 | 0.0008 ± 0.0029 |
| Firmicutes | Eubacteriaceae | Pseudoramibacter _Eubacterium | 0.029 ± 0.0649 | 0.0327 ± 0.0675 |
| Firmicutes | Lachnospiraceae | Other | 0.137 ± 0.2097 | 0.2148 ± 0.5625 |
| Firmicutes | Lachnospiraceae | Unclassified | 4.499 ± 3.0998 | 4.5670 ± 2.8334 |
| Firmicutes | Lachnospiraceae | Anaerostipes | 0.392 ± 0.5054 | 0.2484 ± 0.3940 |
| Firmicutes | Lachnospiraceae | Blautia | 5.512 ± 3.3730 | 5.0652 ± 2.6980 |
| Firmicutes | Lachnospiraceae | Clostridium | 0.248 ± 0.3062 | 0.3474 ± 0.6538 |
| Firmicutes | Lachnospiraceae | Coprococcus | 2.898 ± 2.0151 | 2.1703 ± 1.7916 |
| Firmicutes | Lachnospiraceae | Defluviitalea** | 0.016 ± 0.0201 | 0.0029 ± 0.0064 |
| Firmicutes | Lachnospiraceae | Dorea | 1.252 ± 2.1787 | 0.9839 ± 0.7470 |
| Firmicutes | Lachnospiraceae | Lachnospira | 0.792 ± 1.0619 | 0.7741 ± 1.3514 |
| Firmicutes | Lachnospiraceae | Moryella | 0.001 ± 0.0045 | 0.0022 ± 0.0099 |
| Firmicutes | Lachnospiraceae | Oribacterium* | 0.001 ± 0.0017 | 0.0046 ± 0.0104 |
| Firmicutes | Lachnospiraceae | Roseburia | 3.192 ± 3.4910 | 2.6985 ± 3.0060 |
| Firmicutes | Lachnospiraceae | [Ruminococcus]* | 2.084 ± 2.0740 | 3.4267 ± 2.4854 |
| Firmicutes | Peptococcaceae | Unclassified | 0.003 ± 0.0111 | 0.0006 ± 0.0036 |
| Firmicutes | Peptococcaceae | Peptococcus | 0.029 ± 0.0977 | 0.0244 ± 0.0743 |
| Firmicutes | Peptococcaceae | rc4-4 | 0.005 ± 0.0168 | 0.0043 ± 0.0119 |
| Firmicutes | Peptostreptococcaceae | Peptostreptococcus | 0.001 ± 0.0029 | 0.0008 ± 0.0020 |
| Firmicutes | Peptostreptococcaceae | [Clostridium] | 0.019 ± 0.0993 | 0.0149 ± 0.0872 |
| Firmicutes | Ruminococcaceae | Other | 0.970 ± 1.5549 | 0.8185 ± 1.2608 |
| Firmicutes | Ruminococcaceae | Unclassified | 4.049 ± 2.9464 | 3.1351 ± 3.5722 |
| Firmicutes | Ruminococcaceae | Anaerofilum | 0.003 ± 0.0077 | 0.0047 ± 0.0159 |
| Firmicutes | Ruminococcaceae | Anaerotruncus | 0.032 ± 0.0373 | 0.0532 ± 0.1182 |
| Firmicutes | Ruminococcaceae | Butyricicoccus | 0.460 ± 0.5267 | 0.5612 ± 1.1407 |
| Firmicutes | Ruminococcaceae | Clostridium | 0.006 ± 0.0110 | 0.0064 ± 0.0127 |
| Firmicutes | Ruminococcaceae | Faecalibacterium** | 8.091 ± 5.2759 | 5.0427 ± 4.2863 |
| Firmicutes | Ruminococcaceae | Oscillospira | 3.275 ± 2.2553 | 2.8509 ± 3.2245 |
| Firmicutes | Ruminococcaceae | Ruminococcus | 5.102 ± 5.0913 | 5.6069 ± 7.1556 |
| Firmicutes | Veillonellaceae | Unclassified | 0.001 ± 0.0054 | 0.0013 ± 0.0077 |
| Firmicutes | Veillonellaceae | Acidaminococcus | 0.105 ± 0.2197 | 0.1264 ± 0.3315 |
| Firmicutes | Veillonellaceae | Dialister | 0.406 ± 0.7138 | 0.7160 ± 2.2046 |
| Firmicutes | Veillonellaceae | Megamonas | 2.127 ± 7.2728 | 0.9282 ± 2.3942 |
| Firmicutes | Veillonellaceae | Megasphaera* | 0.386 ± 0.7075 | 1.6762 ± 3.0366 |
| Firmicutes | Veillonellaceae | Mitsuokella | 0.083 ± 0.3626 | 0.2009 ± 1.1741 |
| Firmicutes | Veillonellaceae | Pectinatus | 0.000 ± 0.0000 | 0.0010 ± 0.0061 |
| Firmicutes | Veillonellaceae | Phascolarctobacterium | 1.111 ± 1.1427 | 1.2999 ± 1.4425 |
| Firmicutes | Veillonellaceae | Veillonella | 0.244 ± 0.6799 | 0.3623 ± 0.8359 |
| Firmicutes | [Mogibacteriaceae] | Unclassified | 0.167 ± 0.1588 | 0.1375 ± 0.2071 |
| Firmicutes | [Mogibacteriaceae] | Mogibacterium | 0.011 ± 0.0459 | 0.0015 ± 0.0035 |
| Firmicutes | [Tissierellaceae] | 1-68 | 0.001 ± 0.0062 | 0.0001 ± 0.0006 |
| Firmicutes | [Tissierellaceae] | Anaerococcus | 0.002 ± 0.0092 | 0.0004 ± 0.0020 |
| Firmicutes | [Tissierellaceae] | Finegoldia | 0.001 ± 0.0040 | 0.0043 ± 0.0137 |
| Firmicutes | [Tissierellaceae] | Parvimonas | 0.007 ± 0.0351 | 0.0011 ± 0.0019 |
| Firmicutes | [Tissierellaceae] | Peptoniphilus | 0.002 ± 0.0110 | 0.0022 ± 0.0045 |
| Firmicutes | [Tissierellaceae] | WAL_1855D | 0.003 ± 0.0106 | 0.0004 ± 0.0019 |
| Firmicutes | [Tissierellaceae] | ph2 | 0.001 ± 0.0079 | 0.0003 ± 0.0012 |
| Firmicutes | Unclassified | Unclassified | 0.003 ± 0.0061 | 0.0009 ± 0.0033 |
| Firmicutes | Erysipelotrichaceae | Other | 0.041 ± 0.0811 | 0.1162 ± 0.2589 |
| Firmicutes | Erysipelotrichaceae | Unclassified | 0.291 ± 0.5423 | 0.2601 ± 0.5736 |

| Phylum | Family | Genus | PPI non-users (%) | PPI users (%) |
|---|---|---|---|---|
| Firmicutes | Erysipelotrichaceae | Asteroleplasma | 0.005 ± 0.0280 | 0.0000 ± 0.0000 |
| Firmicutes | Erysipelotrichaceae | Bulleidia | 0.004 ± 0.0162 | 0.0025 ± 0.0038 |
| Firmicutes | Erysipelotrichaceae | Catenibacterium | 0.404 ± 0.8829 | 0.1444 ± 0.4897 |
| Firmicutes | Erysipelotrichaceae | Clostridium | 0.001 ± 0.0071 | 0.0012 ± 0.0047 |
| Firmicutes | Erysipelotrichaceae | Coprobacillus | 0.067 ± 0.1517 | 0.0493 ± 0.1508 |
| Firmicutes | Erysipelotrichaceae | Holdemania | 0.014 ± 0.0162 | 0.0153 ± 0.0273 |
| Firmicutes | Erysipelotrichaceae | [Eubacterium] | 0.560 ± 0.9598 | 0.7808 ± 1.4885 |
| Firmicutes | Erysipelotrichaceae | cc_115 | 0.018 ± 0.0399 | 0.0086 ± 0.0281 |
| Fusobacteria | Fusobacteriaceae | Fusobacterium | 0.655 ± 2.9968 | 1.3257 ± 4.4253 |
| Lentisphaerae | Victivallaceae | Unclassified | 0.037 ± 0.2171 | 0.0021 ± 0.0127 |
| Lentisphaerae | Victivallaceae | Victivallis | 0.012 ± 0.0514 | 0.0061 ± 0.0206 |
| Proteobacteria | Unclassified | Unclassified | 0.031 ± 0.0984 | 0.2226 ± 0.8386 |
| Proteobacteria | Methylobacteriaceae | Methylobacterium | 0.000 ± 0.0000 | 0.0011 ± 0.0065 |
| Proteobacteria | Rhizobiaceae | Agrobacterium | 0.000 ± 0.0000 | 0.0004 ± 0.0022 |
| Proteobacteria | Rhizobiaceae | Rhizobium | 0.000 ± 0.0000 | 0.0018 ± 0.0110 |
| Proteobacteria | Rhodobacteraceae | Rhodobacter | 0.000 ± 0.0000 | 0.0001 ± 0.0005 |
| Proteobacteria | Acetobacteraceae | Unclassified | 0.000 ± 0.0003 | 0.0003 ± 0.0013 |
| Proteobacteria | Sphingomonadaceae | Sphingomonas | 0.000 ± 0.0000 | 0.0003 ± 0.0018 |
| Proteobacteria | Sphingomonadaceae | Sphingopyxis | 0.000 ± 0.0000 | 0.0037 ± 0.0221 |
| Proteobacteria | Alcaligenaceae | Sutterella | 0.404 ± 0.5101 | 0.4572 ± 0.4751 |
| Proteobacteria | Comamonadaceae | Acidovorax | 0.000 ± 0.0000 | 0.0060 ± 0.0361 |
| Proteobacteria | Comamonadaceae | Comamonas | 0.000 ± 0.0000 | 0.0024 ± 0.0146 |
| Proteobacteria | Oxalobacteraceae | Oxalobacter | 0.004 ± 0.0119 | 0.0015 ± 0.0046 |
| Proteobacteria | Desulfovibrionaceae | Other | 0.000 ± 0.0011 | 0.0000 ± 0.0000 |
| Proteobacteria | Desulfovibrionaceae | Unclassified | 0.013 ± 0.0316 | 0.0050 ± 0.0164 |
| Proteobacteria | Desulfovibrionaceae | Bilophila | 0.099 ± 0.1222 | 0.1715 ± 0.1906 |
| Proteobacteria | Desulfovibrionaceae | Desulfovibrio | 0.098 ± 0.2473 | 0.0246 ± 0.0732 |
| Proteobacteria | Campylobacteraceae | Campylobacter | 0.003 ± 0.0106 | 0.0025 ± 0.0052 |
| Proteobacteria | Succinivibrionaceae | Succinatimonas | 0.022 ± 0.0945 | 0.0021 ± 0.0073 |
| Proteobacteria | Enterobacteriaceae | Other | 0.038 ± 0.1553 | 0.0441 ± 0.2241 |
| Proteobacteria | Enterobacteriaceae | Citrobacter | 1.098 ± 3.1215 | 2.7098 ± 7.6726 |
| Proteobacteria | Enterobacteriaceae | Escherichia | 1.691 ± 4.1827 | 2.9503 ± 6.1714 |
| Proteobacteria | Enterobacteriaceae | Klebsiella | 0.592 ± 2.0772 | 0.8817 ± 1.9153 |
| Proteobacteria | Enterobacteriaceae | Morganella | 0.145 ± 0.7557 | 0.3012 ± 1.2225 |
| Proteobacteria | Enterobacteriaceae | Proteus | 0.001 ± 0.0051 | 0.6603 ± 3.3058 |
| Proteobacteria | Enterobacteriaceae | Providencia | 0.050 ± 0.2889 | 0.0000 ± 0.0000 |
| Proteobacteria | Enterobacteriaceae | Serratia | 0.004 ± 0.0106 | 0.2616 ± 0.9554 |
| Proteobacteria | Enterobacteriaceae | Trabulsiella | 0.778 ± 3.7056 | 0.0373 ± 0.1087 |
| Proteobacteria | Pasteurellaceae | Aggregatibacter | 0.000 ± 0.0005 | 0.0010 ± 0.0047 |
| Proteobacteria | Pasteurellaceae | Haemophilus | 0.063 ± 0.1514 | 0.2618 ± 0.7562 |
| Proteobacteria | Moraxellaceae | Psychrobacter | 0.000 ± 0.0000 | 0.0575 ± 0.3447 |
| Proteobacteria | Pseudomonadaceae | Pseudomonas | 0.026 ± 0.1500 | 0.0876 ± 0.4590 |
| Synergistetes | Dethiosulfovibrionaceae | Pyramidobacter | 0.013 ± 0.0513 | 0.0082 ± 0.0230 |
| Synergistetes | Synergistaceae | Unclassified | 0.000 ± 0.0000 | 0.0276 ± 0.1393 |
| Synergistetes | Synergistaceae | Cloacibacillus | 0.020 ± 0.0771 | 0.0169 ± 0.0675 |
| Synergistetes | Synergistaceae | Synergistes | 0.013 ± 0.0639 | 0.0013 ± 0.0076 |
| TM7 | Unclassified | Unclassified | 0.003 ± 0.0050 | 0.0047 ± 0.0064 |
| Tenericutes | Unclassified | Unclassified | 0.030 ± 0.0992 | 0.0055 ± 0.0279 |
| Tenericutes | Unclassified | Unclassified | 0.030 ± 0.1298 | 0.0000 ± 0.0000 |
| Verrucomicrobia | [Cerasicoccaceae] | Unclassified | 0.002 ± 0.0120 | 0.0009 ± 0.0055 |
| Verrucomicrobia | Verrucomicrobiaceae | Akkermansia | 0.613 ± 1.0703 | 0.5840 ± 2.1691 |

Significant differences (*$p$<0.05, **$p$<0.01) between PPI non-users and PPI users.
